# Human Chorionic Gonadotropin Regulates the Smad Signaling Pathway by Antagonizing TGF-β in Giant Cell Tumor of Bone

**DOI:** 10.2174/1574892818666230413082909

**Published:** 2023-12-04

**Authors:** Tangbing Xu, Shenglin Xu, Guangwen Ma, Jun Chang, Chi Zhang, Ping Zhou, Chao Wang, Pengfei Xu, Junjun Yang, Yong Hu, Yunfeng Wu

**Affiliations:** 1 Department of Orthopaedics, The First Affiliated Hospital of Anhui Medical University, Hefei, 230000, Anhui Province, China;; 2 Department of Orthopaedics, Anhui Public Health Clinical Center, Hefei, 230000, Anhui Province, China

**Keywords:** Giant cell tumor of bone (GCTB), human chorionic gonadotropin (HCG), autophagy, epithelial-mesenchymal transition (EMT), smad signaling pathway, TGF-β

## Abstract

***Background*:** Giant cell tumor of bone (GCTB) is a locally aggressive bone tumour aggravated by stromal cell proliferation and metastasis.

***Objective*:** We investigated the mechanism of action of human chorionic gonadotropin (HCG) in mediating GCTB proliferation and invasion.

***Methods*:** The expression of HCG was quantified using quantitative real-time PCR. After the primary stromal cells were isolated and identified, the function of HCG in GCTB was estimated using the cell counting kit-8, flow cytometry, scratch experiment, transwell assay, Western blot, and immunofluorescence. Moreover, the mechanism of HCG was assessed through western blotting.

***Results*:** HCG expression was decreased in clinical tissue samples from patients with GCTB. We validated that HCG repressed stromal cell proliferation, migration, invasion, autophagy, and epithelial-mesenchymal transition (EMT) and promoted cell apoptosis in GCTB. We also verified that HCG repressed the autophagy and EMT of stromal cells through the Smad signaling axis in GCTB. HCG inhibited the transduction of the Smad signaling pathway by restraining the binding of the TGF-β II receptor to ligand Activin A.

***Conclusion*:** HCG restrained the Smad signaling pathway by antagonizing TGF-β signaling in GCTB. HCG may serve as a useful patent to treat GCTB.

## 1 INTRODUCTION

Giant cell tumor of bone (GCTB) is a locally aggressive tumor [1] comprised of multiple cell types, including osteoclast-like multinucleated giant cells, monocytic round-shaped macrophage-like cells, and spindle-shaped fibroblast-like stromal cells [2]. Pathologically, primary stromal cells are the main tumor components of GCTB, and the proliferation and metastasis of stromal cells induce the recurrence of GCTB [3, 4]. Thus, elucidating the molecular mechanisms that promote the growth of stromal cells in GCTB is expected to provide strategies to alleviate GCTB.

Autophagy is a conserved cellular evolutionary process in eukaryotes and can be induced by intracellular or environmental stress [5]. For tumors, autophagy is a “double-edged sword” that can either promote or repress tumor progression, depending on tumor cells, tissue types, and stages [6]. Recently, autophagy has attracted extensive attention for its active role in regulating the interaction between tumor and stromal cells. For instance, Folkerts *et al*. indicated that autophagy in stromal cells produces and supplies nutrients for tumor cells to promote tumorigenesis [7]. Ferraresi *et al*. also clarified that the metabolic interaction between tumor and stromal cells affects the autophagy level of stromal cells in tumors, thus affecting the genesis and development of tumors [8]. However, the exact role of stromal cell autophagy in GCTB remains unclear.

Epithelial-mesenchymal transition (EMT) is the biological process in which epithelial cells transform into cells with a mesenchymal phenotype [9]. In the context of tumors, EMT drives the metastasis and recurrence of tumors and thus plays a pivotal role in tumor progression [10]. Increasing evidence indicates that the occurrence of EMT in stromal cells enhances the invasion and metastasis of tumors [11]. Critically, Chen *et al*. validated that the restraint of EMT in stromal cells alleviates GCTB by repressing cell invasion [12]. Here, considering the key function of EMT in GCTB stromal cells, we conducted a series of experiments to explore the possible mechanism of its occurrence in GCTB stromal cells.

Human chorionic gonadotropin (HCG) is a heterodimeric glycoprotein secreted by placental trophoblast cells and mainly consists of α-and β-subunits [13]. Because of this important and complex molecular form, the role of HCG has been explored in various human diseases, including GCTB. Fitzhugh *et al*. found that the development of GCTB is usually accompanied by the production of β-HCG [14]. Lawless *et al*. demonstrated that β-HCG is abnormally expressed in serum samples of patients with recurrent and metastatic GCTB, implying that β-HCG might be a marker for GCTB [15]. As expected, our current studies indicated that HCG was lowly expressed in GCTB clinical samples and our further functional assays validated that HCG restrained the autophagy and EMT of GCTB stromal cells.

Based on the restraint function of HCG on autophagy and EMT of GCTB stromal cells, we explored the specific molecular mechanism of HCG and the possible related signaling pathways, aiming to provide novel insights and directions for the improvement of GCTB outcomes.

## 2 MATERIALS AND METHODS

### 2.1 Sample Collection

Thirty GCTB patients from the Fourth Affiliated Hospital of Anhui Medical University were included in this study. The tumor tissues were collected from GCTB patients as the experimental group (giant cell tumor of bone), and the normal tissues collected ≥ 3 cm away from the tumors were considered control. Written informed consent was gathered from all patients. This study was approved by the Ethics Committee of the Fourth Affiliated Hospital of Anhui Medical University (PJ-YX2021-018).

### 2.2 Immunohistochemical Analysis

After harvesting the tissue samples from patients with GCTB, the tissues were fixed with 10% formaldehyde, embedded in paraffin blocks, and sectioned into 4-6 µm slices. Subsequently, the slices were incubated with anti-HCG-β (Abcam, ab131170, 1:200 dilution) at 4°C overnight. The slices were then incubated with the secondary antibody (Abcam, ab150077) for approximately 1 h at room temperature (RT). After washing, the sections were stained with hematoxylin and eosin and analyzed using an optical microscope (Olympus BX41, Tokyo, Japan) and photographed for preservation.

### 2.3 Quantitative Real-time PCR

After harvesting GCTB stromal cells with different treatments and GCTB tissues, a TRIzol^®^ reagent (Gibco, Grand Island, NY, USA) was applied to isolate the total RNAs, which were quantified using a NanoDrop 2000 spectrophotometer (ThermoFisher Scientific, WA, USA). Subsequently, the RNAs were reverse transcribed into cDNA using the Revert Aid First Strand cDNA Synthesis kit (Promega Corporation, WI, USA). Immediately after, real-time PCR was conducted on ABI 7500 Real-time PCR Systems (Applied Biosystems, CA, USA) with SYBR Master Mixture (Takara, Kusatsu, Japan). β-actin was applied as an internal reference, and the 2^-∆∆CT^ method was performed to quantify the relative expression of different molecules. Primer sequences are displayed in Table **1**.

### 2.4 Cell Isolation, Culture, and Identification

Clinical tissue samples of GCTB patients were obtained after written informed consent of all patients. After obtaining the fresh GCTB tissues, we isolated primary stromal cells according to previously reported methods [16, 17]. All operations were based on the principles and practice of tumor treatment.

Primary stromal cells were grown in alpha minimum essential medium (α-MEM, Gibco, NY, USA) with 10% fetal calf serum (FCS) and 1% penicillin/streptomycin (ThermoFisher Scientific, WA, USA) at 37°C, 5% CO_2_.

For the identification of primary stromal cells, we performed a qRT-PCR assay to test the expression of CD34 and CD163, which are commonly used markers for GCTB [18, 19]. Additionally, we carried out the Western blot to quantify the CSF1R protein level, which is abnormally expressed in giant cell tumors [20].

### 2.5 Western Blot Assay

GCTB stromal cells with different treatments were collected. Briefly, RIPA lysis buffer (Beyotime, Shanghai, China) was applied for protein extraction from the above cells. Equal amounts of proteins were separated by sodium dodecyl sulfonate-polyacrylamide gel electrophoresis. The above, separated proteins were transferred onto PVDF membranes (Millipore, Billerica, MA, USA) and then blocked with 5% skim milk for 1 hour. The membranes were incubated with primary antibodies: anti-CSF1R (CD115, Invitrogen, Carlsbad, CA, USA), anti-β-actin (Abcam, ab8227, 1:1000), anti-LC3B (Abcam, ab192890, 1:2000), anti-P62 (Abcam, ab109012, 1:10,000), anti-E-cadherin (Abcam, ab40772, 1:10,000), anti-N-cadherin (Abcam, ab76011, 1:5000), anti-AKT (Cell Signaling Technology, #9272, 1:500), anti-p-AKT (Cell Signaling Technology, #4060, 1:500), anti-JNK (Cell Signaling Technology, #9926, 1:1000), anti-p-JNK (Cell Signaling Technology, #9910, 1:1000 dilution), anti-Smad2 (Cell Signaling Technology, #5339, 1:1000), anti-p-Smad2 (Cell Signaling Technology, #18338, 1:1000), anti-Smad3 (Cell Signaling Technology, #9523, 1:1000), anti-p-Smad3 (Cell Signaling Technology, #9520, 1:1000), anti-Activin A (Abcam, ab109300, 1:1000) and anti-GDF-8 (Abcam, ab201954, 1:500) at 4°C overnight. Then, the membranes were incubated with the secondary antibody (Abcam, ab205718, 1:2000) for 1 hour at RT. Antibodies were diluted using PBS (C0221A, Beyotime, Shanghai, China). β-actin was applied as an internal reference. An enhanced chemiluminescence reagent (Millipore, Boston, Massachusetts, USA) was applied for visualization, and ImageJ (Image J v1.8.0, NIH) was conducted to analyze all images.

### 2.6 Different Treatments of Cells

To elucidate HCG effects on GCTB stromal cell growth, the cells were incubated with 0, 2.5, 5, or 10 ng/mL HCG for 24 hours. After the pcDNA-Activin A was transfected into GCTB stromal cells, the cells were then incubated with 10 ng/mL TGF-β2 for 1 day. ActRIIA-Fc can block the binding of Activin A to type II receptors [21]. The GCTB stromal cells were treated with 250 nM ActRIIA-Fc for 1 day.

### 2.7 Cell Counting Kit-8 (CCK-8)

A Cell Counting Kit-8 (Beyotime, Shanghai, China) based on the standard procedure supplied by the manufacturer, was used to test the proliferation of GCTB stromal cells. GCTB stromal cells (1×10^4^) were grown in 96-well plates for nearly 24 hours. Then, 10 μL Cell Counting Kit reagent was added into each well and continued to incubate at 37°C for about 2 hours. A microplate reader was applied to evaluate the absorbance at 450 nm.

### 2.8 Flow Cytometry

GCTB stromal cells (2×10^5^) were grown in 6-well plates for 1 day. After washing, cells were put in annexin V-FITC (ThermoFisher Scientific) for 30 min in the dark, and cells were put in propidium iodide (PI, ThermoFisher Scientific) for 15 min in the dark. The apoptosis of GCTB stromal cells was determined using FACS Canto II flow cytometry (BD Biosciences, Beijing, China).

### 2.9 Scratch Experiment

GCTB stromal cells (5×10^5^) were put in six-well plates for overnight culture. A vertical notch was made in the center of the orifice plate with the tip of a 10 μL pipette until the cells were dispersed to the bottom of the orifice plate. Images of migrating cells were collected at 0 and 48 hours, respectively. The migration region was assessed using the Image J software (Image J v1.8.0, NIH).

### 2.10 Transwell Assay

GCTB stromal cells (5×10^4^) were put in transwell plates (8 mm pore size) and α-MEM with the addition of 10% FCS was applied as a chemoattractant. After incubation for nearly 24 hours, a cotton swab was applied to remove the non-invading cells. Subsequently, the remaining cells were fixed using 4% paraformaldehyde and then stained with hematoxylin. An optical microscope (Olympus BX41, Tokyo, Japan) was applied to count the cells and the cells were photographed for analysis.

### 2.11 Immunofluorescence

GCTB stromal cells were seeded on coverslips and were fixed. The cells were further permeabilized by Triton X-100 solution, followed by the incubation with primary antibodies: anti-LC3B (Abcam, 1 µg/mL) for 60 min at RT. Then the cells were incubated with the secondary antibodies (Abcam). Images were collected through a fluorescence microscope (Olympus IX-71, Tokyo, Japan). The quantitative analysis of the fluorescence signal was carried out by ImageJ software (Image J v1.8.0, NIH).

### 2.12 Cell Transfection

Si-Smad2, pcDNA-Activin A, and the corresponding control (si-NC or pcDNA-NC) were from GenePharma Company (Shanghai, China). Cell transfection was carried out according to the following protocol: GCTB stromal cells (1×10^6^) were grown in a 6-well plate and incubated in culture for about 1 d. According to the manufacturer’s standard protocol, the above synthetic si-Smad2 and pcDNA-Activin A were transfected into GCTB stromal cells using Lipofectamine 2000 (ThermoFisher Scientific).

### 2.13 Statistical Analysis

All statistical analyses were performed using the SPSS 20.0 software. Data are presented as mean ± standard deviation (x ± sd). An Unpaired Student t-test was performed when comparing the differences between the two groups. A one-way ANOVA followed by a Tukey posterior was performed when the differences between more than two groups were compared. A *p*-value less than 0.05 is considered statistically significant.

## 3 RESULTS

### 3.1 HCG is Lowly Expressed in GCTB

**Fig. (1):**
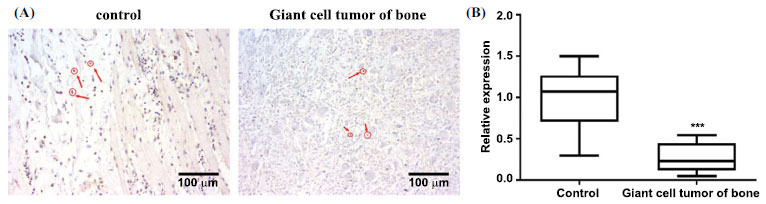
Expression of GCG in giant cell tumor of bone (GCTB). Clinical tumor tissues and adjacent normal tissues of GCTB patients (n = 30) were collected. (**A**) Immunohistochemistry was conducted to test HCG expression in the clinical tissue samples (scale bar: 100 μm); (**B**) Quantitative real-time PCR (qRT-PCR) was conducted to determine HCG expression in the clinical tissue samples. ***p < 0.001 *vs.* control.

To investigate the potential function of HCG in GCTB, we first performed an immunohistochemistry assay for HCG-β to detect its expression in patients with GCTB. As exhibited in Fig. (**1A**), the expression of HCG is reduced in the GCTB group compared to the control group. Similarly, qRT-PCR results demonstrated that HCG was lowly expressed in patients with GCTB (Fig. **1B**). These results support that HCG might play a function in the pathogenesis of GCTB.

### 3.2 Isolation and Identification of GCTB Stromal Cells

**Fig. (2):**
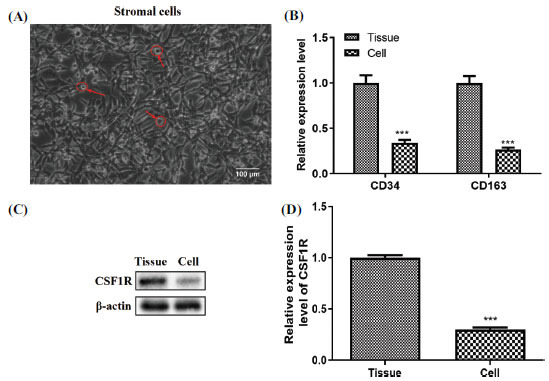
The isolation and identification of GCTB stromal cells. (**A**) Stromal cells were isolated from tissues of GCTB patients (200×); (**B**) The CD34 and CD163 relative levels were determined by qRT-PCR in GCTB tissues and stromal cells; (**C**) CSF1R mRNA and protein levels were quantified using Western blot in GCTB tissues and stromal cells. ***p < 0.001 *vs.* Tissue.

GCTB is a locally aggressive osteoclastic stromal tumor of the bone [22] and the stromal cells of GCTB are primary tumor cells [12]. Stromal cells were isolated from GCTB patients and identified by cell morphology. Cultures of stromal cells contained various slender, spindle-shaped cells (Fig. **2A**). As seen in Fig. (**2B**), stromal cells showed reduced expression of CD34 and CD163 compared to GCTB tissue. Furthermore, the protein level of CSF1R was decreased in stromal cells compared to the GCTB tissues (Fig. **2C**). In general, the above data corroborated the successful separation of GCTB stromal cells from the rest of the tumor tissue.

### 3.3 Effect of HCG on GCTB Stromal Cell Proliferation, Apoptosis, Migration, and Invasion

**Fig. (3):**
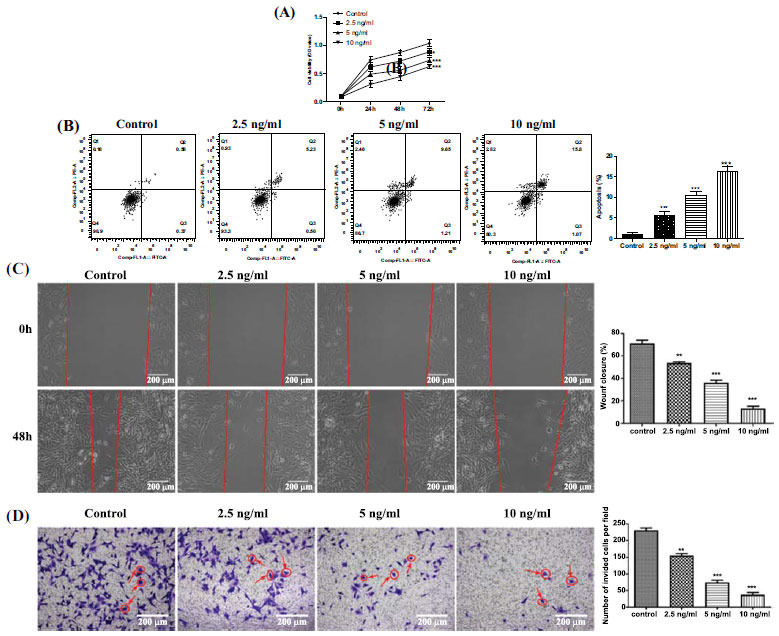
HCG effect on GCTB stromal cell proliferation, apoptosis, migration, and invasion. GCTB stromal cells were incubated with different doses of HCG (0, 2.5, 5, or 10 ng/mL) for 1 day. (**A**) Cell counting kit-8 (CCK-8) was conducted to assess GCTB stromal cell proliferation; (**B**) Flow cytometry assay was carried out to analyze GCTB stromal cell apoptosis; (**C**) A scratch experiment was performed to measure GCTB stromal cell migration ability (100×); (**D**) Transwell migration assay was conducted to characterize the GCTB stromal cell invasion ability (100×). *p < 0.05, **p < 0.01, ***p < 0.001 *vs.* control.

Previous studies demonstrate that restraining the proliferation and metastasis of GCTB stromal cells has the potential to alleviate GCTB [12]. Combined with our previous findings of the low expression of HCG in GCTB (Fig. **1**), we tried to clarify whether HCG regulated GCTB stromal cell proliferation, apoptosis, and migration. We treated GCTB stromal cells with different doses of HCG and examined the different cell indicators. As shown in Fig. (**3A**), the proliferation of GCTB stromal cells was gradually reduced with increased HCG concentration. In contrast, increasing HCG concentration gradually enhanced apoptosis of GCTB stromal cells (Fig. **3B**), and repressed their migration, as measured in scratch assays (Fig. **3C**). Increasing the HCG treatment dose also weakened the invasion ability of GCTB stromal cells, detected in the transwell migration assay (Fig. **3D**). In summary, our data demonstrated that the increased HCG repressed GCTB stromal cell proliferation, migration, and invasion and facilitated cell apoptosis.

### 3.4 HCG Mediates Autophagy in GCTB Stromal Cells

**Fig. (4):**
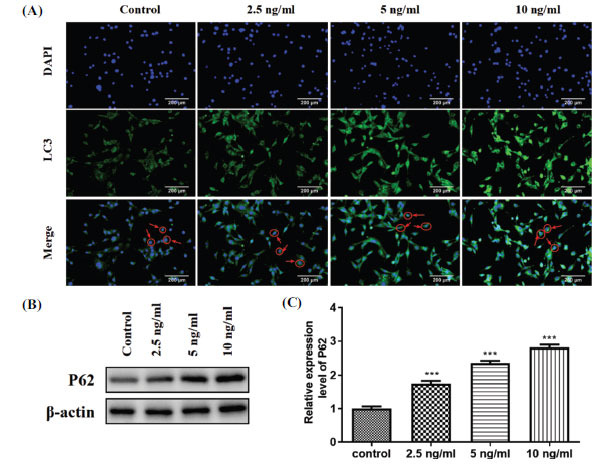
HCG influence on the autophagy in GCTB stromal cells. The GCTB stromal cells were incubated with different doses of HCG (0, 2.5, 5, or 10 ng/mL) for 1 day. (**A**) Immunofluorescence assay was carried out to assess the expression of autophagy marker protein LC3 (400×); (**B**) Western blot was conducted to test autophagy marker protein P62 protein level; (**C**) The quantitative analysis of P62 proteins. ***p < 0.001 *vs.* control.

Increasing evidence suggests that autophagy has a pivotal function in maintaining the survival of stromal cells [23, 24]. We attempt to elucidate the effect of HCG on autophagy in GCTB stromal cells. LC3 and P62 are widely used as autophagy marker proteins [25]. Immunofluorescence and immunoblotting showed that the expression levels of LC3 (Fig. **4A**) and P62 (Fig. **4B**) were increased in GCTB stromal cells treated with HCG. The quantitative analysis of the P62 protein is displayed in Fig. (**4C**). In conclusion, treatment of HCG repressed autophagy in GCTB stromal cells.

### 3.5 HCG Inhibits the EMT of GCTB Stromal Cells

**Fig. (5):**
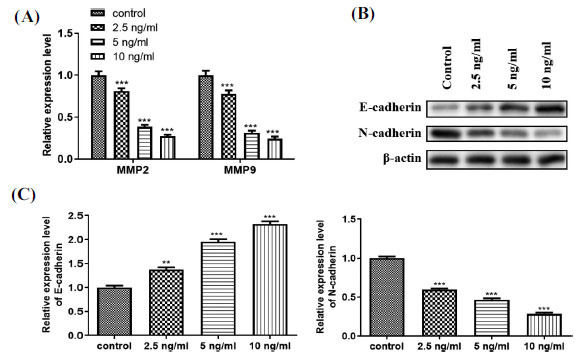
HCG effect on the epithelial-mesenchymal transition (EMT) of GCTB stromal cells. GCTB stromal cells were incubated with 0, 2.5, 5, or 10 ng/mL HCG for 1 d. (**A**) The relative expressions of the invasion and migration-related genes MMP-2 and MMP-9 were determined by qRT-PCR; (**B**) The protein levels of EMT-associated proteins E-cadherin and N-cadherin were quantified through Western blot; (**C**) The quantitative analysis of E-cadherin and N-cadherin protein levels. **p < 0.01, ***p < 0.001 *vs.* control.

Epithelial cells acquire the ability to migrate through EMT, and EMT exerts a key role in the occurrence of cancer metastasis [9]. To investigate whether HCG is involved in regulating EMT in GCTB stromal cells, the cells were treated with different doses of HCG. As exhibited in Fig. (**5A**), the relative expression of invasion- and migration-related genes MMP-2 and MMP-9 were decreased with the increased HCG treatment dose (Fig. **5A**). As expected, the detection of EMT-associated proteins revealed that the protein level of E-cadherin was gradually up-regulated with the increased HCG dose and N-cadherin was gradually down-regulated (Fig. **5B**), suggesting that HCG restrained the EMT of GCTB stromal cells. The quantitative results for the E-cadherin and N-cadherin proteins are shown in Fig. (**5C**). Overall, the above results verify that HCG represses EMT in GCTB stromal cells.

### 3.6 HCG Mediates the Autophagy and EMT of GCTB Stromal Cells Through the Smad Pathway

**Fig. (6):**
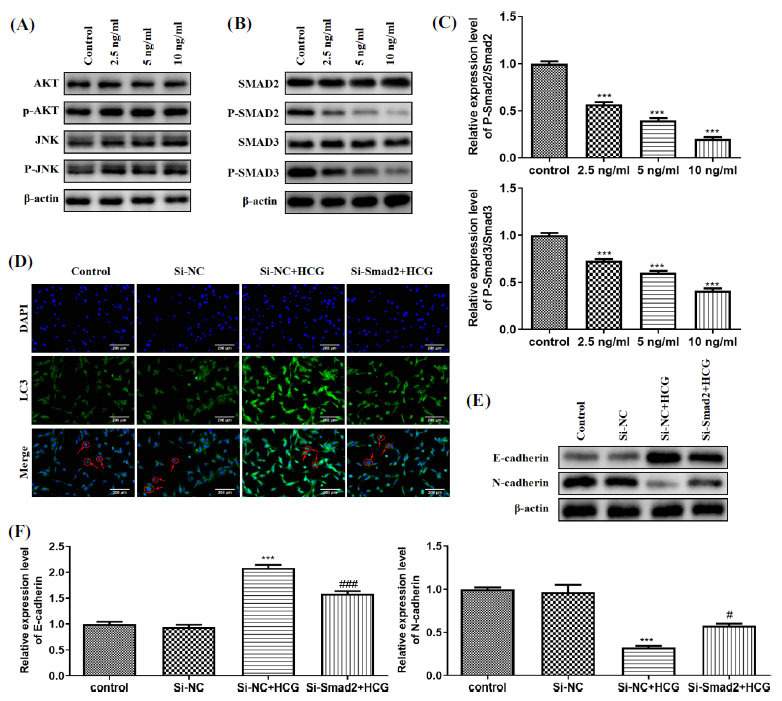
HCG affects the autophagy and EMT of GCTB stromal cells through the Smad signaling axis. GCTB stromal cells were incubated with 0, 2.5, 5, or 10 ng/mL HCG for 1 d. (**A**) Western blot analysis was conducted to assess the protein levels of Akt, p-Akt, JNK, and p-JNK; (**B**) Detection of the protein levels of Smad2, p-Smad2, Smad3 and p-Smad3 by Western blot; (**C**) The quantitative results of p-Smad2/Smad2 and p-Smad3/Smad3. si-Smad2 or si-NC was transfected into GCTB stromal cells, and the cells were then incubated with 10 ng/mL HCG; (**D**) Immunofluorescence was conducted to test the LC3 expression (400×); (**E**) Detection of protein levels of E-cadherin and N-cadherin by Western blot; (**F**) The quantitative analysis of E-cadherin and N-cadherin protein levels. ***p < 0.001 *vs.* control or si-NC. ^#^*p* < 0.05, ^###^*p* < 0.001 *vs.* si-NC + HCG.

Subsequently, we explore how HCG regulates autophagy and EMT in GCTB stromal cells. In recent years, the pivotal functions of signaling pathways in GCTB have gradually attracted widespread attention, and the AKT, and JNK signaling pathways are interrelated to autophagy [26, 27]. Thus, we tried to clarify whether Akt and JNK signaling pathways regulated autophagy in GCTB stromal cells after HCG treatment. As displayed in Fig. (**6A**), the protein levels of Akt, p-Akt, JNK, and p-JNK did not change significantly with increasing HCG dose, suggesting that Akt and JNK signaling pathways do not regulate the autophagy of GCTB stromal cells after HCG treatment. The Smad signaling axis also has a critical function in autophagy and EMT [28]. As shown in Fig. (**6B**), the protein levels of p-Smad2 and p-Smad3 decreased after HCG treatment. Quantitative analysis of p-Smad2/Smad2 and p-Smad3/Smad3 proteins showed that the expression levels of p-Smad2/Smad2 and p-Smad3/Smad3 proteins decreased gradually with the increase of HCG concentration (Fig. **6C**). Based on these findings, we transfected si-Smad2 into GCTB stromal cells and treated the cells with HCG. By immunofluorescence, compared with the si-NC + HCG group, the transfection of si-Smad2 reduced the inhibitory effect of HCG on autophagy (Fig. **6D**). Furthermore, compared with the si-NC + HCG group, the forced reduction in Smad2 expression lessened E-cadherin and raised N-cadherin levels (Figs. **6E** and **F**), suggesting that the transfection of si-Smad2 weakened the restraint of HCG on EMT. These results indicate that HCG mediates the autophagy and EMT of GCTB stromal cells by regulating the Smad signaling axis.

### 3.7 HCG Regulates the Smad Pathway by Repressing the Binding of TGF-β to its Receptor

**Fig. (7):**
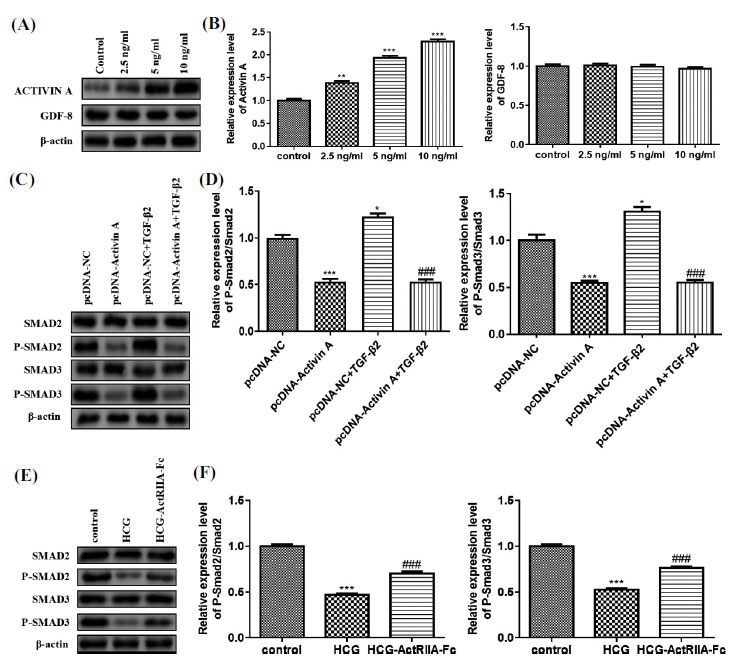
HCG influences the Smad signaling axis by regulating the binding of TGF-β to its receptor. GCTB stromal cells were incubated with 0, 2.5, 5, or 10 ng/mL HCG for 1 d. (**A**) Western blot was carried out to quantify Activin A and GDF-8 protein levels; (**B**) The quantitative analysis of Activin A and GDF-8 protein levels. After the pcDNA-Activin A was transfected into GCTB stromal cells, the cells were then incubated with 10 ng/mL TGF-β2 for 1 d; (**C**) Detection of Smad2, p-Smad2, Smad3, and p-Smad3 protein levels by Western blot; (**D**) The quantitative results of p-Smad2/Smad2 and p-Smad3/Smad3. The GCTB stromal cells were incubated with HCG (10 ng/mL) and/or ActRIIA-Fc (250 nM) for 1 d; (**E**): Smad2, p-Smad2, Smad3, and p-Smad3 protein levels were determined by Western blot; (**F**): The quantitative analysis of p-Smad2/Smad2 and p-Smad3/Smad3. *p < 0.05 *vs.* pcDNA-NC. **p < 0.01 *vs.* control. ***p < 0.001 *vs.* control, pcDNA-NC. ^###^*p* < 0.001 *vs.* HCG, pcDNA-NC + TGF-β2.

Studies authenticate that the Smad signaling pathway is regulated by the TGF-β type II receptor, and the TGF-β type II receptor can be activated by binding to ligands Activin A and GDF-8 [29-31]. Following treatment with increasing concentrations of HCG, the protein level of Activin A in the GCTB stromal cells was gradually up-regulated, whereas GDF-8 did not significantly change (Figs. **7A** and **B**). Thus, Activin A was selected as the main ligand to be analyzed in subsequent studies. We then transfected pcDNA-Activin A into GCTB stromal cells and treated the cells with TGF-β2. As exhibited in Fig. (**7C**), compared with the pcDNA-NC group, TGF-β2 treatment elevated p-Smad2 and p-Smad3, while Activin A overexpression decreased p-Smad2 and p-Smad3 protein levels (Fig. **7D**). ActRIIA-Fc has been found to block the binding of Activin A to type II receptors [21]. After the GCTB stromal cells were treated with HCG and/or ActRIIA-Fc, ActRIIA-Fc elevated p-Smad2 and p-Smad3 (Fig. **7E**); quantitative results of p-Smad2/Smad2 and p-Smad3/Smad3 are shown in Fig. (**7F**). Taken together, the above results corroborated that HCG regulates the Smad signaling axis by restraining the binding of TGF-β2 to its receptor.

## 4 DISCUSSION

Several previous studies have validated the abnormal expression of HCG in GCTB, bringing new hope for the exploration of potential biomarkers for GCTB [15, 32]. As expected, our research also observed that HCG expression was decreased in clinical tissue samples from patients with GCTB, implying that HCG might be involved in the initiation of GCTB. For the functional study of HCG, we validated that HCG repressed the metastasis of stromal cells in GCTB. This observation was similar to the conclusion from a previous study that the proliferation and metastasis of stromal cells promote GCTB development [33]. Furthermore, our in-depth study revealed that HCG mediated the autophagy and EMT of stromal cells through the Smad signaling pathway and regulated this signaling pathway by restraining the binding of TGF-β to its receptor. This is the first research to clarify the mechanism of HCG in GCTB.

A previous study demonstrated that GCTB is not a malignant tumor and that stromal cells cannot proliferate indefinitely [4]. Therefore, to elucidate the molecular mechanism of the GCTB *in vitro* assay, we isolated and identified the primary stromal cells for subsequent functional assays, which demonstrated that HCG restrained stromal cell proliferation, migration, and invasion and promoted apoptosis in GCTB. This finding was similar to the previously reported results [34]. Numerous studies have shown that the autophagy and EMT of stromal cells play important regulatory functions in tumors [35], and our study also validated that HCG repressed the autophagy and EMT of stromal cells in GCTB, as shown previously.

GCTB metastasis is a complex process involving multiple key and influential signaling pathways. For instance, Chen *et al*. reported that targeting the AKT signaling pathway alleviates GCTB by restraining GCTB stromal cell proliferation and metastasis [12]. Mak *et al*. determined the inactivation of the JNK signaling pathway represses the up-regulation of MMP-13 in GCTB stromal cells to regulate GCTB progression [36]. Our findings suggest that HCG presents a dose-dependent inhibition of GCTB stromal cells proliferation, migration, and invasion, and increased apoptosis rates. Our study further attempted to explore whether HCG mediated the autophagy and EMT of GCTB stromal cells through the AKT or JNK signaling pathways. Our data confirmed that HCG did not significantly change p-AKT and p-JNK expression, implying that HCG does not mediate the autophagy and EMT of GCTB stromal cells through the Akt or JNK signaling pathways. Furthermore, accumulated evidence suggests that the Smad signaling pathway induced by transforming the growth factor-beta (TGF-β) family restrains tumor progression by inducing autophagy and EMT in tumor cells [37, 38]. Similarly, our results validated that HCG decreased p-Smad2 and p-Smad3 and the knockdown of Smad2 reduced the autophagy and EMT of GCTB stromal cells induced by HCG.

Smad protein transduction results from signals from the TGF-β receptors, and TGF-β-type II receptors phosphorylate Smad2 and Smad3 by binding ligands Activin A and growth differentiation factor-8 (GDF-8) and thus lead to intracellular signaling [39-41]. In the current study, we sought to clarify the specific mechanism by which HCG restrained the binding of TGF-β2 and its receptor and our data demonstrated that blocking the binding of Activin A to type II receptors restrained the regulation of HCG on the Smad signaling pathway. This result further enriched the content of this study.

## CONCLUSION

In general, our study validated that HCG repressed the autophagy and EMT of stromal cells through the Smad signaling axis in GCTB and HCG inhibited the transduction of the Smad signaling pathway by restraining the binding of TGF-β2 to its receptor.

## CURRENT AND FUTURE DEVELOPMENT

Our study provides new insights and ideas for the treatment of GCTB, which is of great significance. However, our study has some limitations. For example, further experimental validation of GCTB stromal cells proliferation, migration, invasion, and apoptosis by HCG is still lacking. In addition, the signaling pathways mediating EMT ability of GCTB stromal cells still need further investigation.

## Figures and Tables

**Table 1:** Primer sequences used in qRT-PCR.

Gene Name	Primer Sequence (5’-3’)
HCG	Forward: GCAGGGGACGCACCAAGGA
	Reverse: CACGCGGGTCATGGTGGG
CD34	Forward: ACCAGAGCTATTCCCAAAAGACC
	Reverse: TGCGGCGATTCATCAGGAAAT
CD163	Forward: TCAGACACTATCCCCGTGCA
	Reverse: GGCGAAGTTGACCACTCCC
CSF1R	Forward: GCAGTACCACCATCCACTTGTA
	Reverse: GTGAGACACTGTCCTTCAGTGC
MMP-2	Forward: TGATGGCATCGCTCAGATCC
	Reverse: GGCCTCGTATACCGCATCAA
MMP-9	Forward: GGACAAGCTCTTCGGCTTCT
	Reverse: TCGCTGGTACAGGTCGAGTA
β-actin	Forward: AGC GAG CAT CCC CCA AAG TT
	Reverse: GGG CAC GAA GGC TCA TCA TT

## Data Availability

The data and supportive information is available within the article.

## LIST OF ABBREVIATIONS

EMT: Epithelial-Mesenchymal Transition
GCTB: Giant Cell Tumor of Bone
HCG: Human Chorionic Gonadotropin
RT: Room Temperature
